# Gut microbiota-derived metabolites and EVs-mediated signaling in type 2 diabetes mellitus

**DOI:** 10.3389/fcell.2026.1862369

**Published:** 2026-07-08

**Authors:** Xi-Peng Chen, Jia-Qi Xu, Nan-Nan Zhang, Qiang Huang, Tao-Hong Zhu, Chao He

**Affiliations:** Department of Central Laboratory, Fourth Affiliated Hospital of Jiangsu University, Zhenjiang, China

**Keywords:** extracellular vesicles, gut microbiota, immunometabolism, microbial metabolites, type 2 diabetes mellitus

## Abstract

Type 2 diabetes mellitus is characterized by systemic insulin resistance, chronic low-grade inflammation, and progressive metabolic dysfunction. Increasing evidence identifies the gut microbiota as a central regulator of host immunometabolism through a diet–microbiota–host axis. Gut microbiota-derived metabolites, including short-chain fatty acids, bile acids, branched-chain amino acids, and trimethylamine N-oxide, integrate endocrine signaling, intracellular metabolic pathways, and inflammatory responses across intestinal and systemic compartments, thereby shaping glucose homeostasis and metabolic balance. Diet acts as an upstream determinant by modulating microbial composition and metabolic activity. Beyond soluble metabolites, extracellular vesicles have emerged as an additional mode of intercellular communication. Vesicles derived from diet or microbiota carry bioactive cargos such as proteins, lipids, and small RNAs, enabling the transfer of functional signals that may influence both microbial ecology and host immunometabolic processes. This review summarizes metabolite-dependent and vesicle-mediated signaling pathways and highlights how these interconnected mechanisms position the gut microbiota as a signaling hub linking dietary inputs to host cellular regulation. This framework provides a conceptual basis for microbiota-targeted strategies in the prevention and treatment of type 2 diabetes.

## Introduction

Type 2 diabetes mellitus (T2DM) is characterized by systemic insulin resistance and progressive β-cell dysfunction. Metabolic stress and chronic low-grade inflammation induced by overnutrition and physical inactivity contribute to impaired insulin signaling and disease progression (Donath and Shoelson, 2011; Hotamisligil, 2017). Emerging evidence identifies the gut microbiota as a central regulator of host immunometabolism through a microbiota–metabolite-immunometabolic axis linking dietary to immune and metabolic regulation (Donald and Finlay, 2023; Zmora et al., 2017; Machado et al., 2025).

Gut microbial composition and function are altered in individuals with T2DM, accompanied by disrupted host metabolic and immune homeostasis. Microbiota-derived signals can be organized into interrelated categories, including microbial metabolites (e.g., short-chain fatty acids and indole derivatives), microbiota-dependent host metabolites (e.g., bile acids and trimethylamine N-oxide), and host–microbiota co-metabolic signatures (e.g., branched-chain amino acids) (Jin et al., 2025; Mei et al., 2024). These signaling pathways integrate microbial metabolism with host inflammatory and endocrine responses, contributing to inflammatory response and insulin resistance in T2DM. As an upstream determinant, diet shapes these processes by modulating microbial composition and metabolic activity, thereby influencing the abundance of microbiota-derived signals. In addition, diet-derived extracellular vesicles (EVs) may contribute to this regulatory network by delivering bioactive cargos, particularly microRNAs, with the potential to modulate microbial and host gene expression (Liu et al., 2025; Shalvina et al., 2026).

**FIGURE 1:**
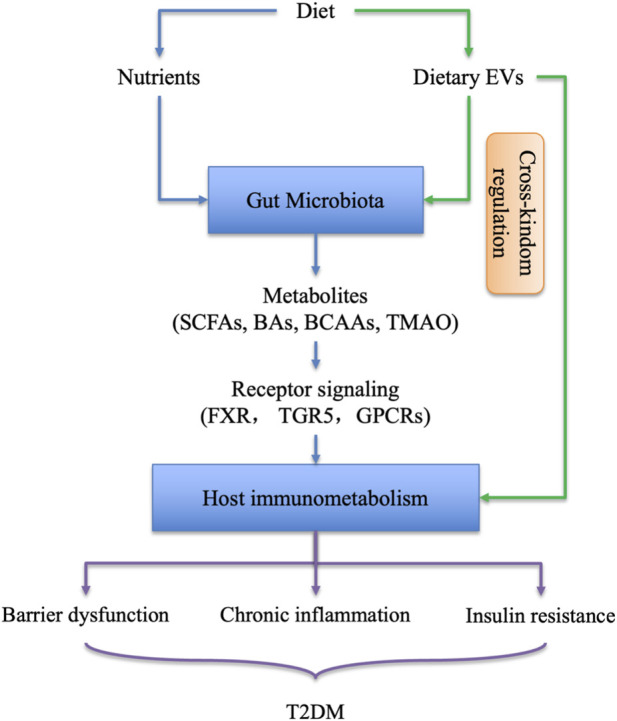
Diet–microbiota–host signaling networks in T2DM. Diet shapes gut microbial metabolism, generating metabolites (SCFAs, BAs, BCAAs, TMAO) that regulate host immunometabolism. In parallel, extracellular vesicles (EVs), derived from diet or microbiota, deliver cargos such as microRNAs that modulate microbial function and host responses, potentially *via* cross-kingdom regulation. These pathways converge on epithelial barrier function, inflammation, and insulin sensitivity, contributing to T2DM. SCFAs: short-chain fatty acids; BAs: bile acids; BCAAs: branched-chain amino acids; TMAO: trimethylamine N-oxide; FXR: farnesoid X receptor; TGR5: Takeda G protein–coupled receptor 5; GPCRs: G protein–coupled receptors.

This review focuses on microbiota-derived functional signals in T2DM, encompassing metabolite-mediated pathways and vesicle-associated cargos, and discusses how diet shapes these networks to link gut microbiota with host immunometabolic regulation (Figure 1).

## Microbiota-derived metabolites in host immunometabolic regulation

Gut microbiota convert dietary substrates and host-derived compounds into a wide spectrum of bioactive signals through microbial metabolism and host–microbiota co-metabolic processes (Jin et al., 2025). These metabolite-mediated signals operate across intestinal and systemic compartments to regulate host immunometabolic homeostasis (Baars et al., 2024; Jin et al., 2025).

At the intestinal barrier, microbial metabolites are essential for maintaining mucosal immune homeostasis, and disruption of this balance contributes to chronic low-grade inflammation, a key driver of T2DM. Short-chain fatty acids (SCFAs), primarily produced through fermentation of dietary fiber by anaerobic bacteria such as Faecalibacterium and Roseburia, exert immune modulatory effects through both epigenetic and receptor-mediated mechanisms. At the epigenetic level, SCFAs such as butyrate and propionate, enter immune cells *via* monocarboxylate transporters and inhibit class I/IIa histone deacetylases (HDACs), thereby increasing the accessibility of regulatory regions of the FOXP3 locus, enhancing transcription factor binding, including STAT5 and NFAT, and promoting regulatory T cell (Treg) differentiation (Furusawa et al., 2013; Pham et al., 2024). At the receptor signaling level, SCFAs activate G protein–coupled receptors, including GPR41 and GPR43, to modulate macrophage polarization and induce a tolerogenic dendritic cell phenotype characterized by reduced antigen presentation and co-stimulatory capacity, thereby further attenuating effector T cell responses (Liu et al., 2024; Pham et al., 2024). In addition to immune modulation, SCFAs are functionally coupled to epithelial barrier integrity. They promote the expression of tight junction proteins, including zonula occludens-1 (ZO-1) and occludin, through G protein–coupled receptor signaling (e.g., GPR41/43) (Peng et al., 2007; Ma et al., 2012) and enhance mucus production by upregulating mucin gene expression (e.g., MUC2) (Baars et al., 2024), thereby limiting microbial translocation-induced inflammation in T2DM. In parallel, tryptophan-derived metabolites, such as indole derivatives, activate the aryl hydrocarbon receptor (AhR) in intestinal epithelial cells and promote its nuclear translocation, activating IL-22–dependent STAT3 signaling (Zelante et al., 2013). This pathway supports epithelial regeneration and enhances barrier integrity by promoting epithelial proliferation, tight junction maintenance, and antimicrobial peptide production (Zelante et al., 2013; Agus et al., 2018).

Beyond the gut, microbiota-derived metabolites enter the systemic circulation and target insulin-responsive tissues, including liver, adipose tissue, and skeletal muscle. These metabolic organs play central roles in maintaining glucose and lipid homeostasis, and their dysregulation represents a key pathological feature of T2DM (Baars et al., 2024). Secondary bile acids (BAs), generated through gut microbiota–mediated transformation of primary bile acids synthesized in the host liver, function as endocrine-like signaling molecules *via* farnesoid X receptor (FXR) and Takeda G protein–coupled receptor 5 (TGR5). FXR signaling, primarily active in the liver and intestine, regulates BAs synthesis, hepatic glucose production (HGP), and lipid metabolism through fibroblast growth factor 19 (FGF19)-mediated feedback suppression of gluconeogenic programs (Liu et al., 2024; Gao et al., 2026; Schlicht et al., 2025). Accordingly, impaired FXR signaling has been implicated in dysregulated HGP and systemic insulin resistance in T2DM. In contrast, TGR5 activation in intestinal L cells and peripheral metabolic tissues, including adipose tissue and skeletal muscle, promotes glucagon-like peptide-1 (GLP-1) secretion and enhances energy expenditure through cAMP–protein kinase A (PKA)-dependent signaling pathways (Liu et al., 2024; Hernández-Montoliu et al., 2023). Other than BAs–mediated endocrine signaling, additional microbiota-associated metabolites further contribute to systemic metabolic dysregulation in T2DM. Elevated circulating branched-chain amino acids (BCAAs) reflect perturbations in host–microbiota co-metabolism of amino acids and interfere with intracellular insulin signaling through activation of mTORC1 and S6K1-mediated inhibitory phosphorylation of insulin receptor substrate-1 (IRS1) (Shah et al., 2024). In parallel, BCAAs accumulation is associated with incomplete fatty acid oxidation and accumulation of acylcarnitine intermediates, which promote mitochondrial stress and activate pro-inflammatory signaling pathways, including JNK and NF-κB, in metabolic tissues (White et al., 2018). Moreover, trimethylamine N-oxide (TMAO), a host–microbiota co-metabolite generated from dietary choline and carnitine through microbial production of trimethylamine (TMA) followed by hepatic oxidation (Tang et al., 2013), contributes to metabolic dysfunction by promoting NF-κB–dependent inflammatory signaling and disrupting cholesterol homeostasis, including impairment of reverse cholesterol transport (RCT) and modulation of bile acid synthesis (Wang et al., 2011; Tang et al., 2013; Seldin et al., 2016).

Collectively, microbiota-derived metabolites function as key signaling mediators that integrate endocrine regulation, intracellular metabolic pathways, and inflammatory responses. Through coordinated effects on these interconnected processes, they shape systemic glucose homeostasis, lipid metabolism, and chronic low-grade inflammation, which are central features of T2DM pathophysiology. This metabolite-driven signaling represents a fundamental mechanism by which the gut microbiota influences host metabolic status (Table 1).

**TABLE 1:** Microbiota-derived metabolites in T2DM: sources, mechanisms, and functional relevance.

Metabolite	Source	Receptors and signaling	Major functions	Relevance to T2DM
SCFAs	Diet fiber	GPCRs	Promote treg differentiation Anti-inflammation Enhance intestinal barrier	Insulin sensitivity Chronic low-grade inflammation
Secondary BAs	Primary BAs	FXR TGR5	Regulate BAs synthesis Regulate glucose metabolism	Insulin resistance
BCAAs	Diet protein	mTORC1 JNK/NF-κb	Regulate mitochondrial stress Regulate inflammation	Insulin resistance
TMAO	TMA	NF-κb	Promote inflammation Disrupt RCT Alter bile acid synthesis	Metabolic dysfunction
Tryptophan catabolites	Tryptophan	AhR STAT3	Enhance epithelial barrier	Maintain gut barrier integrity
LPS	G^−^ bacteria	TLR4/NF-κb	Systemic inflammation	Chronic low-grade inflammation

SCFAs: short-chain fatty acids; BAs: bile acids; BCAAs: branched-chain amino acids; TMAO: trimethylamine N-oxide; LPS, lipopolysaccharide; TMA: trimethylamine; G^−^: Gram-negative; GPCRs: G protein–coupled receptors; FXR: farnesoid X receptor; TGR5: Takeda G protein–coupled receptor 5; mTORC1: mechanistic target of rapamycin complex 1; JNK: c-Jun N-terminal kinase; NF-κB, nuclear factor kappa B; AhR: aryl hydrocarbon receptor; STAT3: signal transducer and activator of transcription 3; TLR4: Toll-like receptor 4; RCT: reverse cholesterol transport.

## Extracellular vesicle-mediated immunometabolic signals in T2DM

Diet is a major environmental determinant of T2DM, with dietary patterns strongly associated with the risk and progression of metabolic dysfunction (Baars et al., 2024; Guo et al., 2025). Beyond direct effects on host metabolism, diet also influences disease progression indirectly. By regulating substrate availability, diet modulates not only microbial composition but also their metabolism, thereby influencing host immunometabolic homeostasis, as described above. Beyond soluble metabolites, extracellular vesicles (EVs) represent a structurally and functionally distinct mode of communication of diet–microbiota–host communication (Liu et al., 2025; Marquez-Paradas et al., 2025; Verbunt et al., 2024), with emerging relevance to T2DM.

Plant-derived EVs, a substantial fraction of dietary vesicles, are enclosed by lipid bilayer membranes that protect their molecular cargos from enzymatic degradation, enabling persistence within the gastrointestinal tract. Plant-derived EVs influence host immunometabolism through two interconnected mechanisms: by modulating the composition and metabolic activity of the gut microbiota, and by directly acting on intestinal epithelial cells to regulate barrier integrity and inflammatory tone, processes that are frequently disrupted in T2DM (Shalvina et al., 2026; Zhang et al., 2024). These effects are largely mediated by vesicle-associated cargos. Among these cargos, small RNAs, particularly plant-derived microRNAs, have been proposed to mediate cross-kingdom gene regulation *via* sequence-specific interactions with target transcripts (Zhang et al., 2012), thereby enabling post-transcriptional regulation in both microbiota and host (Buck et al., 2014). Mechanistically, these microRNAs typically promote target transcript cleavage or translational repression through RNA-induced silencing complex (RISC)-mediated pathways (O'Brien et al., 2018), suggesting a conserved silencing machinery that may, under certain conditions, extend regulatory potential across biological kingdoms. Notably, plant microRNAs are characterized by 2′-O-methylation at their 3′termini, which enhances resistance to exonuclease-mediated degradation and, together with vesicular encapsulation, confers increased stability in the gastrointestinal environment, which supports their persistence during digestion and potential interactions with gut microbes and intestinal epithelial cells. In the context of T2DM-associated epithelial barrier disruption and microbiota dysbiosis, EV-associated RNA signaling may constitute an underappreciated dietary regulatory layer of metabolic homeostasis. Their *in vivo* functional relevance, however, remains to be fully established.

In parallel, microbiota-derived EVs constitute a functionally active component of this diet–microbiota–host communication network. These vesicles carry proteins, lipids, and nucleic acids that are closely associated with the metabolic and physiological state of gut bacteria and can directly interact with host epithelial and immune cells. Microbiota-derived EVs represent a complementary component of the signaling network involved in immunometabolic regulation in T2DM. For example, *Bacteroides* fragilis-derived EVs contain polysaccharide A, which promotes regulatory T cell differentiation and suppresses inflammatory responses, thereby supporting insulin-sensitive immune states (Shen et al., 2012). Similarly, Akkermansia muciniphila-derived EVs enhance intestinal barrier integrity and improve metabolic phenotypes in preclinical models, including improved glucose tolerance and reduced adipose tissue inflammation (Chelakkot et al., 2018; Depommier et al., 2019). These effects have been associated with attenuation of endotoxin-driven inflammatory signaling, particularly the LPS–TLR4–NF-κB axis, which contributes to inflammation-associated insulin resistance in T2DM (Cani et al., 2007; Kaparakis-Liaskos and Ferrero, 2015).

Collectively, EVs constitute a distinct communication system within the diet–microbiota–host axis. Beyond soluble metabolites, EVs enable the transfer of structurally protected and functionally diverse cargos across dietary, microbial, and host compartments, thereby supporting stable inter-kingdom signaling. Within this framework, plant-derived EVs and microbiota-derived EVs represent complementary pathways linking diet to microbial ecology and host immunometabolic regulation. This EV-mediated signaling network may be particularly relevant in T2DM, where intestinal barrier dysfunction and chronic inflammation converge. However, the quantitative contribution and *in vivo* relevance of EV-based cross-kingdom signaling in human metabolic disease remain to be fully defined.

## Perspectives

Despite substantial progress in understanding gut microbiota–host interactions, current therapeutic strategies for T2DM remain largely focused on host-directed pharmacological approaches, with limited exploitation of microbiota and diet-derived signals.

Microbiota-derived metabolites represent well-established functional mediators of host immunometabolism, while EVs provide an additional, structurally protected mode of inter-kingdom communication. These two complementary signaling modalities form a multilayered regulatory network linking diet, microbiota, and host immunometabolic responses. Importantly, these pathways are not merely descriptive but provide actionable therapeutic opportunities. Metabolite-centered interventions, such as dietary fiber modulation or BAs receptor targeting, have already demonstrated clinical potential, whereas EV-mediated signaling—particularly involving diet-derived and microbial-derived EVs—remains an emerging but largely untapped therapeutic Frontier (Frias et al., 2023).

Therefore, further studies are needed to delineate the causal contribution, tissue specificity, and translational feasibility of EV-based regulations. Integrating metabolite-based and vesicle-based strategies may ultimately enable a more precise therapeutic framework for T2DM.

## References

[B1] Agus A. Planchais J. Sokol H. (2018). Gut microbiota regulation of tryptophan metabolism in health and disease. Cell Host Microbe 23, 716–724. 10.1016/j.chom.2018.05.003 29902437

[B2] Baars D. P. Fondevila M. F. Meijnikman A. S. Nieuwdorp M. (2024). The central role of the gut microbiota in the pathophysiology and management of type 2 diabetes. Cell Host Microbe 32, 1280–1300. 10.1016/j.chom.2024.07.017 39146799

[B3] Buck A. H. Coakley G. Simbari F. McSorley H. J. Quintana J. F. Le Bihan T. (2014). Exosomes secreted by nematode parasites transfer small RNAs to Mammalian cells and modulate innate immunity. Nat. Commun. 5, 5488. 10.1038/ncomms6488 25421927 PMC4263141

[B4] Cani P. D. Amar J. Iglesias M. A. Poggi M. Knauf C. Bastelica D. (2007). Metabolic endotoxemia initiates obesity and insulin resistance. Diabetes 56, 1761–1772. 10.2337/db06-1491 17456850

[B5] Chelakkot C. Choi Y. Kim D.-K. Park H. T. Ghim J. Kwon Y. (2018). Akkermansia muciniphila-derived extracellular vesicles influence gut permeability through the regulation of tight junctions. Exp. Mol. Med. 50, e450. 10.1038/emm.2017.282 29472701 PMC5903829

[B6] Depommier C. Everard A. Druart C. Plovier H. Van Hul M. Vieira-Silva S. (2019). Supplementation with Akkermansia muciniphila in overweight and Obese human volunteers: a proof-of-concept exploratory study. Nat. Med. 25, 1096–1103. 10.1038/s41591-019-0495-2 31263284 PMC6699990

[B7] Donald K. Finlay B. B. (2023). Early-life interactions between the microbiota and immune system: impact on immune system development and atopic disease. Nat. Rev. Immunol. 23, 735–748. 10.1038/s41577-023-00874-w 37138015

[B8] Donath M. Y. Shoelson S. E. (2011). Type 2 diabetes as an inflammatory disease. Nat. Rev. Immunol. 11, 98–107. 10.1038/nri2925 21233852

[B9] Frias J. P. Lee M. L. Carter M. M. Ebel E. R. Lai R.-H. Rikse L. (2023). A microbiome-targeting fibre-enriched nutritional formula is well tolerated and improves quality of life and haemoglobin A1c in type 2 diabetes: a double-blind, randomized, placebo-controlled trial. Diabetes Obes. Metab. 25, 1203–1212. 10.1111/dom.14967 36594522

[B10] Furusawa Y. Obata Y. Fukuda S. Endo T. A. Nakato G. Takahashi D. (2013). Commensal microbe-derived butyrate induces the differentiation of colonic regulatory T cells. Nature 504, 446–450. 10.1038/nature12721 24226770

[B11] Gao G. Wang E. Yang G. Gao Y. Zhao Z. Zhao Y. (2026). 20(S/R)-ginsenoside Rh1 improves type 2 diabetes via gut microbiota-modulated bile acid signaling and FXR/TGR5-dependent GLP-1 enhancement. Biomed. and Pharmacother. 195, 118997. 10.1016/j.biopha.2026.118997 41546911

[B12] Guo H. Pan L. Wu Q. Wang L. Huang Z. Wang J. (2025). Type 2 diabetes and the multifaceted Gut-X axes. Nutrients 17, 2708. 10.3390/nu17162708 40871736 PMC12389143

[B13] Hernández-Montoliu L. Rodríguez-Peña M.-M. Puig R. Astiarraga B. Guerrero-Pérez F. Virgili N. (2023). A specific gut microbiota signature is associated with an enhanced GLP-1 and GLP-2 secretion and improved metabolic control in patients with type 2 diabetes after metabolic Roux-en-Y gastric bypass. Front. Endocrinol. (Lausanne) 14, 1181744. 10.3389/fendo.2023.1181744 37916149 PMC10616869

[B14] Hotamisligil G. S. (2017). Inflammation, metaflammation and immunometabolic disorders. Nature 542, 177–185. 10.1038/nature21363 28179656

[B15] Jin J. Yang X. Feng R. Ye M. Xu H. Wang J. (2025). Gut microbiota‐derived metabolites orchestrate metabolic reprogramming in diabetic cardiomyopathy: mechanisms and therapeutic frontiers. FASEB J. 39, e71004. 10.1096/fj.202501579RR 40899744 PMC12406765

[B16] Kaparakis-Liaskos M. Ferrero R. L. (2015). Immune modulation by bacterial outer membrane vesicles. Nat. Rev. Immunol. 15, 375–387. 10.1038/nri3837 25976515

[B17] Liu R. Wang J. Zhao Y. Zhou Q. Yang X. Gao Y. (2024). Study on the mechanism of modified Gegen Qinlian decoction in regulating the intestinal flora-bile acid-TGR5 axis for the treatment of type 2 diabetes mellitus based on macro genome sequencing and targeted metabonomics integration. Phytomedicine 132, 155329. 10.1016/j.phymed.2023.155329 38853123

[B18] Liu Z. Yin R. Tian J. (2025). Extracellular vesicles: mechanisms and prospects in type 2 diabetes and its complications. Front. Endocrinol. 15, 1521281. 10.3389/fendo.2024.1521281 40212823 PMC11983144

[B19] Ma X. Fan P. X. Li L. S. Qiao S. Y. Zhang G. L. Li D. F. (2012). Butyrate promotes the recovering of intestinal wound healing through its positive effect on the tight junctions. J. Anim. Sci. 90 (Suppl. 4), 266–268. 10.2527/jas.50965 23365351

[B20] Machado J. L. P. Schaan A. P. Mamede I. Fernandes G. R. (2025). Gut microbiota and type 2 diabetes associations: a meta-analysis of 16S studies and their methodological challenges. Front. Microbiomes 4, 1506387. 10.3389/frmbi.2025.1506387 41852438 PMC12993587

[B21] Marquez-Paradas E. Torrecillas-Lopez M. Barrera-Chamorro L. Del Rio-Vazquez J. L. Gonzalez-de La Rosa T. Montserrat-de La Paz S. (2025). Microbiota-derived extracellular vesicles: current knowledge, gaps, and challenges in precision nutrition. Front. Immunol. 16, 1514726. 10.3389/fimmu.2025.1514726 40051622 PMC11882860

[B22] Mei Z. Wang F. Bhosle A. Dong D. Mehta R. Ghazi A. (2024). Strain-specific gut microbial signatures in type 2 diabetes identified in a cross-cohort analysis of 8,117 metagenomes. Nat. Med. 30, 2265–2276. 10.1038/s41591-024-03067-7 38918632 PMC11620793

[B23] O’Brien J. Hayder H. Zayed Y. Peng C. (2018). Overview of MicroRNA biogenesis, mechanisms of actions, and circulation. Front. Endocrinol. (Lausanne) 9, 402. 10.3389/fendo.2018.00402 30123182 PMC6085463

[B24] Peng L. He Z. Chen W. Holzman I. R. Lin J. (2007). Effects of butyrate on intestinal barrier function in a Caco-2 cell monolayer model of intestinal barrier. Pediatr. Res. 61, 37–41. 10.1203/01.pdr.0000250014.92242.f3 17211138

[B25] Pham N. H. T. Joglekar M. V. Wong W. K. M. Nassif N. T. Simpson A. M. Hardikar A. A. (2024). Short-chain fatty acids and insulin sensitivity: a systematic review and meta-analysis. Nutr. Rev. 82, 193–209. 10.1093/nutrit/nuad042 37290429 PMC10777678

[B26] Schlicht K. Pape L. Rohmann N. Knappe C. Epe J. Geisler C. (2025). Prediabetes and type 2 diabetes but not obesity are associated with alterations in bile acid related gut microbe-microbe and gut microbe-host community metabolism. Gut Microbes 17, 2474143. 10.1080/19490976.2025.2474143 40045464 PMC11901388

[B27] Seldin M. M. Meng Y. Qi H. Zhu W. Wang Z. Hazen S. L. (2016). Trimethylamine N-Oxide promotes vascular inflammation through signaling of mitogen-activated protein kinase and nuclear Factor-κB. J. Am. Heart Assoc. 5, e002767. 10.1161/JAHA.115.002767 26903003 PMC4802459

[B28] Shah H. Gannaban R. B. Haque Z. F. Dehghani F. Kramer A. Bowers F. (2024). BCAAs acutely drive glucose dysregulation and insulin resistance: role of AgRP neurons. Nutr. Diabetes 14, 40. 10.1038/s41387-024-00298-y 38844453 PMC11156648

[B29] Shalvina A. Paul N. A. Cummins S. F. Eamens A. L. (2026). Plant extracellular vesicles with complex molecular cargo: a cross-kingdom conduit for MicroRNA-Directed RNA silencing. Genes 17, 52. 10.3390/genes17010052 41595472 PMC12840914

[B30] Shen Y. Giardino Torchia M. L. Lawson G. W. Karp C. L. Ashwell J. D. Mazmanian S. K. (2012). Outer membrane vesicles of a human commensal mediate immune regulation and disease protection. Cell Host Microbe 12, 509–520. 10.1016/j.chom.2012.08.004 22999859 PMC3895402

[B31] Tang W. H. W. Wang Z. Levison B. S. Koeth R. A. Britt E. B. Fu X. (2013). Intestinal microbial metabolism of phosphatidylcholine and cardiovascular risk. N. Engl. J. Med. 368, 1575–1584. 10.1056/NEJMoa1109400 23614584 PMC3701945

[B32] Verbunt J. Jocken J. Blaak E. Savelkoul P. Stassen F. (2024). Gut-bacteria derived membrane vesicles and host metabolic health: a narrative review. Gut Microbes 16, 2359515. 10.1080/19490976.2024.2359515 38808455 PMC11141482

[B33] Wang Z. Klipfell E. Bennett B. J. Koeth R. Levison B. S. DuGar B. (2011). Gut flora metabolism of phosphatidylcholine promotes cardiovascular disease. Nature 472, 57–63. 10.1038/nature09922 21475195 PMC3086762

[B34] White P. J. McGarrah R. W. Grimsrud P. A. Tso S.-C. Yang W.-H. Haldeman J. M. (2018). The BCKDH kinase and phosphatase integrate BCAA and lipid metabolism *via* regulation of ATP-Citrate lyase. Cell Metab. 27, 1281–1293.e7. 10.1016/j.cmet.2018.04.015 29779826 PMC5990471

[B35] Zelante T. Iannitti R. G. Cunha C. De Luca A. Giovannini G. Pieraccini G. (2013). Tryptophan catabolites from microbiota engage aryl hydrocarbon receptor and balance mucosal reactivity via Interleukin-22. Immunity 39, 372–385. 10.1016/j.immuni.2013.08.003 23973224

[B36] Zhang L. Hou D. Chen X. Li D. Zhu L. Zhang Y. (2012). Exogenous plant MIR168a specifically targets Mammalian LDLRAP1: evidence of cross-kingdom regulation by microRNA. Cell Res. 22, 107–126. 10.1038/cr.2011.158 21931358 PMC3351925

[B37] Zhang S. Wang Q. Tan D. E. L. Sikka V. Ng C. H. Xian Y. (2024). Gut-liver axis: potential mechanisms of action of food-derived extracellular vesicles. J. Extracell. Vesicles 13, e12466. 10.1002/jev2.12466 38887165 PMC11183959

[B38] Zmora N. Bashiardes S. Levy M. Elinav E. (2017). The role of the immune system in metabolic health and disease. Cell Metab. 25, 506–521. 10.1016/j.cmet.2017.02.006 28273474

